# Is Muscle Architecture Different in Athletes with a Previous Hamstring Strain? A Systematic Review and Meta-Analysis

**DOI:** 10.3390/jfmk7010016

**Published:** 2022-01-31

**Authors:** Eleftherios Kellis, Chrysostomos Sahinis

**Affiliations:** Laboratory of Neuromechanics, Department of Physical Education and Sport Sciences at Serres, Aristotle University of Thessaloniki, 62100 Serres, Greece; sachinisc@phed-sr.auth.gr

**Keywords:** hamstring, injury, review, fascicle, tendon, return to play

## Abstract

Hamstring strains are a frequent injury in sports and are characterized by a high recurrence rate. The aim of this review was to examine the muscle and tendon architecture in individuals with hamstring injury. A systematic literature search in four databases yielded eleven studies on architecture following injury. Differences in the fascicle length (FL), pennation angle (PA) and muscle size measures (volume, thickness and physiological cross-sectional area) at rest were not significantly different between the previously injured limb and the contralateral limb (*p* > 0.05). There was moderate evidence that biceps femoris long head (BFlh) FL shortening was greater during contraction in the injured compared to the contralateral limb. The BFlh FL was smaller in athletes with a previous injury compared to uninjured individuals (*p* = 0.0015) but no differences in the FL and PA of other muscles as well as in the aponeurosis/tendon size were observed (*p* > 0.05). An examination of the FL of both leg muscles in individuals with a previous hamstring strain may be necessary before and after return to sport. Exercises that promote fascicle lengthening of both injured and uninjured leg muscles may be beneficial for athletes who recover from a hamstring injury.

## 1. Introduction

Hamstring injuries are frequent in athletes and may lead to extensive absence from sports [1]. Further, a considerable number of athletes (up to 30%) who recover from such an injury, sustain the same injury again [1,2]. This explains why a history of previous injury is one of the few factors that is associated with an increased risk for injury [3]. Hence, management of hamstring injuries is important for reducing recurrent incidents.

When athletes return to play, they may still experience some strength and flexibility deficits [4,5,6,7,8]. Following a controlled rehabilitation program, for example, athletes experience a 9.6% deficit in peak torque relative to the uninjured limb immediately after return to play, which is minimized 6 months later [6]. There are also reports that athletes maintain deficits in maximum and explosive strength for an even longer period of time (18–33 months after injury) [9,10]. A recent metanalysis concluded that strength and flexibility deficits continue even after return to play, although this effect depends on the method that is used to quantify muscle strength [8]. The question which arises is why these deficits remain after return to play.

Muscle strength can be influenced by several factors such as neural activation [11] and the morphology and mechanical properties of the muscle and tendon [12]. Acute hamstring injury with evident structural damage is accompanied by edema and fluid concentration, which gradually leads to the formation of a scar in the injured area [13,14,15,16]. Further, a recent metanalysis concluded that there is moderate evidence that individuals with a previous strain show a lower activation of the injured muscle during eccentric contractions compared to the uninjured limb [17]. Such events may influence the size and morphology of the muscle–tendon unit. However, the evidence is inconsistent as some studies have found that hamstring injury influences muscle architecture such as the fascicle length (FL) and angle of pennation (PA) [18,19,20] but others have reported opposite results [21,22]. One might also expect that injury influences muscle size parameters, such as muscle volume, thickness (MT) or cross-sectional area (CSA) but the results are conflicting [6,22,23,24]. Finally, early studies have suggested that injury leads to changes in aponeuroses and tendon geometry [25] but recent studies have reported opposite results [24].

Understanding the acute and chronic effects of hamstring strain on muscle and tendon architecture can provide important information about the management of such injuries before and after return to play [26]. In the absence of pre-injury measurements, for example, the function of the contralateral (non-injured) muscle may serve as a target for the rehabilitation of the injured muscle. For this reason, it is important to understand if injury affects the architecture of the injured leg only. Another question is whether individuals with a history of hamstring strain show differences in muscle–tendon architecture compared with controls. If this is the case, then screening athletes for muscle–tendon architecture deficiencies may be useful for the prevention of recurrent as well as first-time injuries. In addition, since there are various measures of size and architecture and four different individual hamstring muscles, there is a need for a detailed examination of the evidence for each one of them. To the best of our knowledge, alterations in the muscle and tendon properties after injury have not been systematically reviewed. Therefore, the aim of this study was to systematically review the research evidence on hamstring muscle and tendon architecture characteristics of previously injured hamstrings. We examined, firstly, the differences in architecture between the injured and the non-injured contralateral leg in individuals with a previous hamstring injury, and secondly, the differences in architecture between previously strain-injured limbs and uninjured controls.

## 2. Methods

### 2.1. Literature Search and Study Selection

This systematic review was performed according to the Preferred Reporting Items (PRISMA) for Systematic Reviews and Meta-Analyses. The review protocol was prospectively registered on the database for Open Science Framework (OSF: 10.17605/OSF.IO/QPJW8). A comprehensive search strategy was conducted to identify all relevant articles without any time limits in four electronic databases (PubMed, Medline, Cochrane library and Scopus) up until 17 October 2021 using the PICO framework. This included population: athletes or recreational active individuals; investigated condition: hamstring strain injury; comparison condition: comparison of injured and uninjured groups and injured with healthy contralateral leg; and outcome of interest: hamstring muscle architectural characteristics. The electronic search was performed independently by the two authors (E.K. and C.S.) to mitigate the probability of study selection bias. The following search syntax was used in all databases: (Hamstring * OR Semitendinosus OR Semimebranosus OR “Biceps femoris” OR “Posterior Thigh”) AND (Injur * OR Strain * OR Tear OR Rupture *) AND (“muscle Morphology *” OR “Cross * sectional area” OR CSA OR “pennation angle” OR “fiber length” OR “muscle volume *” OR fascicle OR “tendon morphology *” OR “aponeurosis morphology *”). Further, secondary searches were conducted to ensure the identification of all the relevant studies by (1) performing forward citation tracking of the included studies through Scopus and Google Scholar, and (2) screening the reference list from the included studies and from previous related systematic reviews. After duplicates were removed, relevant articles identified through the search strategy had their title and abstract independently screened for eligibility by two authors (E.K. and C.S.) in accordance with the inclusion and exclusion criteria. Full-texts that met the inclusion criteria were assessed independently by the two authors. Any discrepancies in the included and excluded studies were resolved through discussion and consensus.

### 2.2. Data Extraction

Data extraction was performed independently by the two authors, using a customized data extraction Microsoft Excel spreadsheet (Microsoft Excel v 2016, One Microsoft Way Redmond, Washington, DC, USA). Data extracted from each article comprised: (1) authors and year of publication, (2) details regarding participants’ characteristics (such as sample size, sex, age, sport and level of partition), (3) injury characteristics (injured muscle and severity), (4) the study’s methodological characteristics (muscle under examination, study design, medical imaging methodology), (5) FL, PA, muscle length (ML), muscle and tendon aponeurosis variables (CSA, thickness, volume, length or width), and (6) primary outcome measures (i.e., means and standard deviations) for all reported muscle architectural characteristics of injured and healthy participants. 

### 2.3. Elibility Criteria

The inclusion and the exclusion criteria were determined prior to the search to ensure objectivity during the study identification procedure. In particular, studies that satisfied the following criteria were included in the review: (1) publications in international English-language peer-reviewed journal, (2) studies that included participants that had suffered a HSI and had returned to normal activity level, (3) studies that explored the differences in hamstrings architecture between individuals with HIS and a control group or the contralateral uninjured leg. Reviews, case and brief reports, letters to editors, theses and conference abstracts were excluded.

### 2.4. Quality Assessment

The methodological quality of the included studies was examined by two reviewers (E.K. and C.S.) independently using a modified version of the Downs and Black Checklist [8,27]. In particular, the original checklist includes 27 items; however, items 4, 8, 9, 13, 15, 17, 19, 22, 23, 24 and 26 were initially excluded, and item 27 was modified as these items were designed only for intervention studies while this review contains only retrospective studies. Additionally, items 28 and 29 were added to explore the methodological quality of the injury diagnosis and the rehabilitation procedure [8,28]. The scale had a maximum score of 20 as items 5, 28 and 29 could take values of 2 or 1 or 0 while the remaining items were scored either as 1 or 0. A quality index (QI) was also calculated by dividing the sum of scores by the number of items. A score ≥70% was considered to indicate a low risk of bias while a score <70% was considered to indicate a high risk of bias.

### 2.5. Data Analysis

When two or more experimental studies provided data for the same outcome measure, we used meta-analyses to compare, first, the two legs of athletes with a previous injury, and second, the injured leg of the injured group and the corresponding leg(s) of the control group. Μeta-analysis was conducted using the ‘metafor’ package in R (v 4.0.2; R Core Team, Vienna, Austria, https://www.r-project.org/ date assessed on 15 December 2021). We applied the random-effects model with a restricted maximum likelihood (REML) method to better account for potential methodological or statistical heterogeneity of the included studies. The standardized mean difference (SMD) and 95% confidence intervals (CIs) were calculated. The magnitudes of the SMDs were interpreted as: “trivial” (<0.20), “small” (0.20–0.39), “medium” (0.40–0.59), “large” (0.60–0.80), and “very large” (>0.80) [29]. The heterogeneity of the included studies were assessed using the I2 statistic and it was interpreted as “low” (<50%), “moderate” (50–75%), or “high” (>75%) [30]. Funnel plot asymmetry was not examined given that there were less than ten studies included in the meta-analyses [31]. The statistical significance threshold was set at *p* < 0.05. 

In cases where only one study provided data for a specific outcome variable, metanalysis was not possible owing to the limited data availability and the small number of studies (n = 1). In this case, a best evidence synthesis was conducted [32]. The strength of evidence was ranked according to the following criteria:

1. Strong evidence: consistent results in two or more low risk of bias studies with generally consistent findings in ≥75% of studies.
2. Moderate evidence: provided by one low risk of bias study and/or two or more studies with high risk of bias study and by generally consistent results across all studies (≥75% of the studies reported consistent findings).
3. Limited evidence: provided by single-study findings from high risk of bias study.
4. Conflicting evidence: inconsistent findings in multiple studies (<75% of the studies reported consistent findings).
5. No evidence: no studies (randomized controlled trials or non-randomized controlled trials) available for assessment.

## 3. Results

### 3.1. Search Results

**Figure 1:**
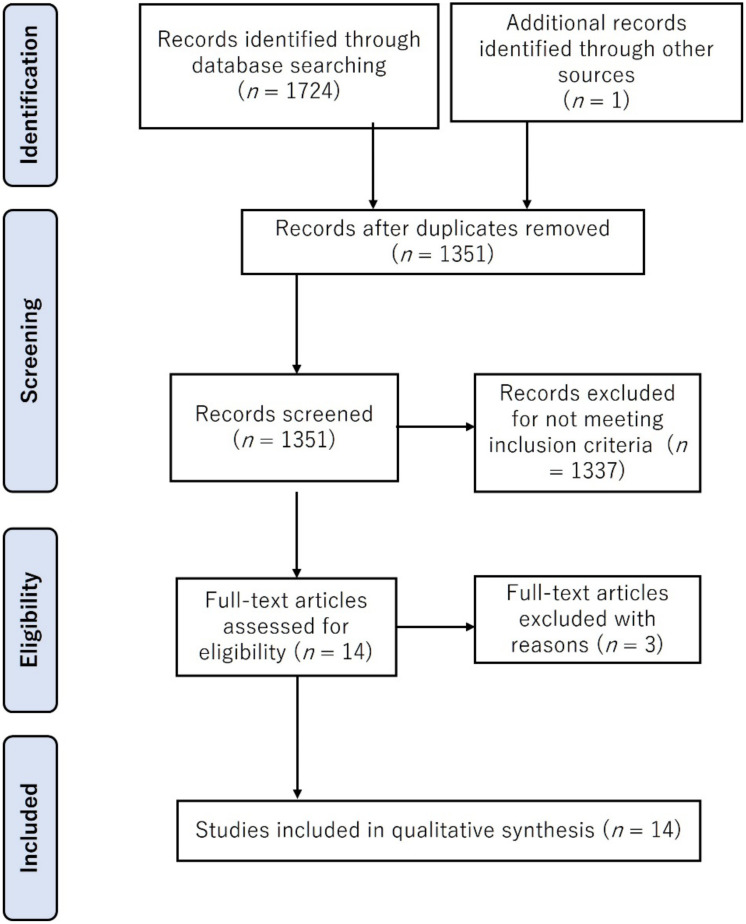
Flow diagram outlining steps for study inclusion/exclusion in this review.

The database searches identified 1724 potentially relevant published studies (Figure 1). From these, 373 duplicate articles were removed while after title and abstract screening, 1337 documents were excluded as they did not meet the inclusion criteria. Of the remaining 14 articles, 3 additional studies were excluded in the full-text analysis. Hence, 11 articles were retained for further analysis.

### 3.2. Risk of Bias Assessment

The results for the risk of bias assessment of each included article are presented in Table 1. The methodological quality scores of the included studies ranged from 9 (45%) to 17 (85%), with a mean score of 13.6 (68.1%). Seven studies were rated as having a low risk of bias, while the remaining four studies were rated as having a high risk of bias. As evidenced in Table 1, studies received a high-risk bias score for items 11 and 12, which address whether participants are representative of the population, item 27, which indicates a lack of power calculations, and item 25, which examines the absence or presence of adequate adjustment for confounding factors in the analyses.

### 3.3. Description of Studies

The detailed characteristics of the included studies are summarized in Table 2. A total of 294 participants (males n = 285 and females n = 9) were examined. The sample size ranged from 6 [35] to 80 participants [19] with an age range from 19.25 ± 2.40 to 28.6 ± 5.2 years.

Eleven studies on muscle architecture compared the injured with the uninjured limb [6,18,19,20,21,22,23,24,33,34,35] while five studies compared the injured individuals with controls [18,19,20,21,24].

Of the included studies, five examined the FL [18,19,20,21,22], four studies examined muscle PA [18,20,21,22], MT [18,20,21,35] and muscle volume [6,22,24,34] while the CSA was examined by three studies [22,24,36]. Nine studies examined the BFlh, four studies the ST, two studies the BFsh and one investigated the SM. Finally, two studies examined the hamstring architecture by considering all individual muscle heads as one muscle [34,36], and aponeurosis morphology [24] and tendon volume [23] were studied by one study each.

### 3.4. Injured vs. Uninjured Limb

**Figure 2:**
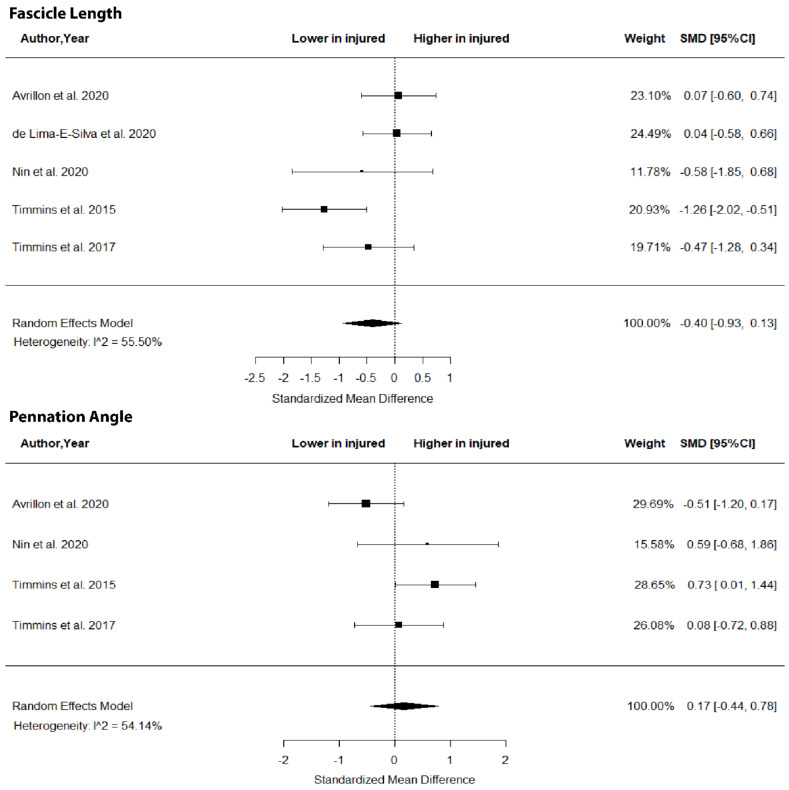
Forest plot of biceps femoris long head fascicle length and angle of pennation in injured versus contralateral limb of athletes with a previous hamstring strain.

The metanalysis showed no differences in BFlh FL (SMD = −0.40; 95% CI −0.93 to 0.1; *p* > 0.05; I^2^ = 0%) and PA (SMD = 0.17; 95% CI −0.44 to 0.78; *p* > 0.05; I^2^ = 54.15%) between legs in previously injured individuals (Figure 2).

**Figure 3:**
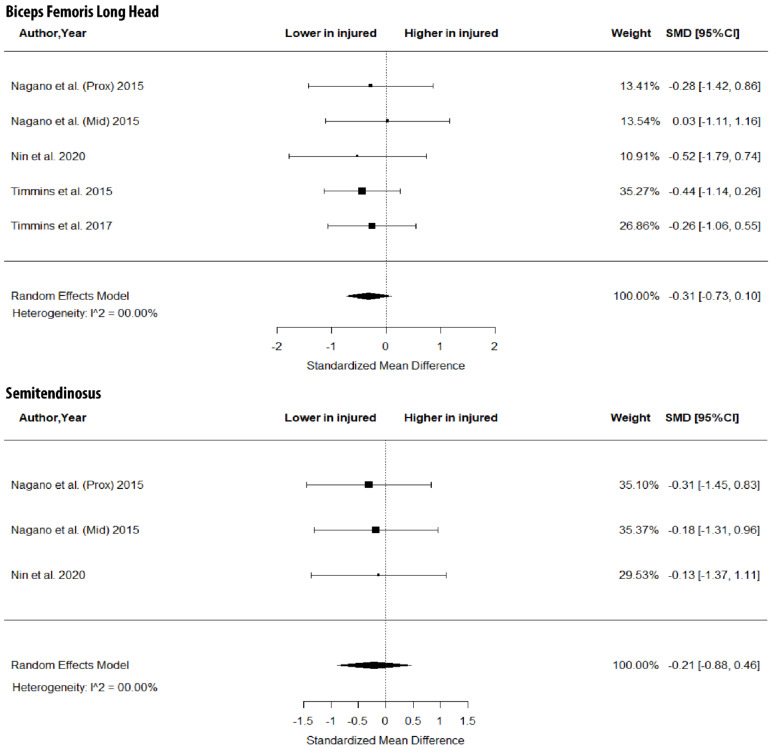
Forest plot of muscle thickness of biceps femoris long head and semitendinosus in injured versus the contralateral limb of athletes with a previous hamstring strain.

**Figure 4:**
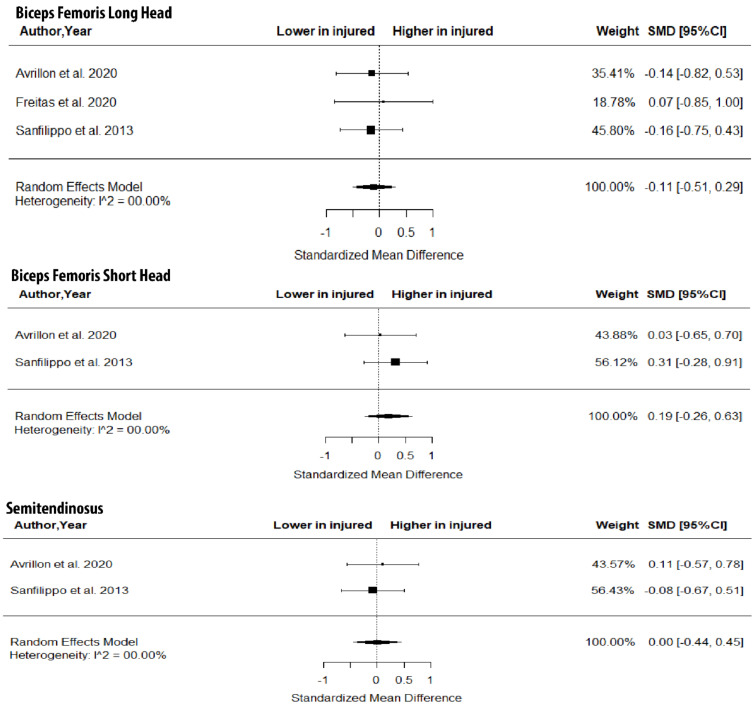
Forest plot of differences in volume of biceps femoris long and short heads and semitendinosus between individuals with a previous hamstring strain and controls.

As shown in Figure 3, there were no between-leg differences in BFlh MT (SMD = −0.31; 95% CI −0.73 to 0.10; *p* > 0.05; I^2^ = 0%) and ST MT (SMD = −0.21; 95% CI −0.88 to 0.46; *p* > 0.05; I^2^ = 0%). Further, the metanalysis showed non-significant between-limb differences in the volume of the BFlh (SMD = −0.11; 95% CI −0.51 to 0.29; *p* > 0.05; I^2^ = 0%), BFsh (SMD = 0.19; 95% CI −0.26 to 0.63; *p* > 0.05; I^2^ = 0%) and ST (SMD = 0.00; 95% CI −0.44 to 0.45; *p* > 0.05; I^2^ = 0%) (Figure 4).

The results from the best-evidence analysis are presented in Table 3. There was moderate (SM, BFsh) or limited (ST) evidence that the FL was not different between the injured and contralateral leg in athletes with a previous injury. Moderate evidence also showed that BFlh FL/muscle length does not differ between the contralateral and injured leg. In contrast, BFlh FL and BFlh FL/MT at contraction were lower in the injured leg compared with the contralateral one. 

For PA, most comparisons between the two limbs showed no differences. In only one case, moderate evidence suggested a lower PA of the BFlh in the injured compared to the contralateral limb (Table 3). For CSA and volume variables, no differences were observed. In only one case, there was moderate evidence that the BFlh/hamstring physiological CSA ratio was lower in the injured compared to the contralateral leg. Moderate evidence indicates that the aponeurosis area, width or volume does not differ between the injured and contralateral limb, but limited evidence for a greater BFlh tendon volume in the injured leg was found.

### 3.5. Injured vs. Controls

**Figure 5:**
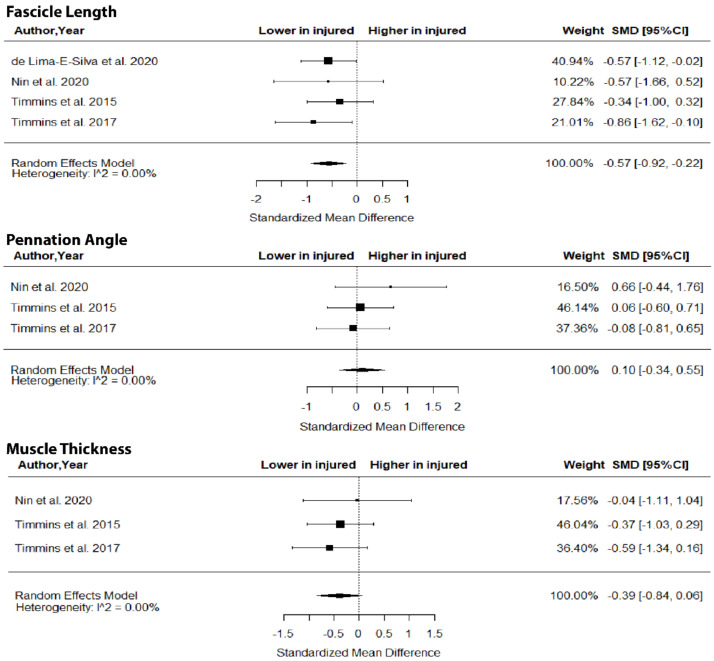
Forest plot of differences in fascicle length, pennation angle and muscle thickness of biceps femoris long head between individuals with a previous hamstring strain and controls.

The metanalysis showed that BFlh FL was significantly lower in previously injured athletes compared to controls (SMD = −0.57; 95% CI −0.92 to −0.22; *p* = 0.0015; I^2^ = 0%), but no group differences in PA (SMD = 0.10; 95% CI −0.34 to 0.55; *p* > 0.05; I^2^ = 0%) and MT (SMD = −0.39; 95% CI −0.84 to 0.06; *p* > 0.05; I^2^ = 0%) were found (Figure 5).

The results from the best-evidence analysis for group comparisons are presented in Table 4. When comparing injured athletes with controls, the ST FL (limited evidence) and BFlh FL/muscle length (moderate evidence) were lower in injured athletes. Limited or moderate evidence indicated that there are no group differences in FL at contraction, PA, muscle and tendon/aponeurosis morphology variables.

## 4. Discussion

Our metanalysis indicated no between-limb differences in FL, PA or MT in individuals with a hamstring strain. The best evidence synthesis analysis showed that there is moderate evidence of a lower BFlh FL at contraction and greater tendon volume in the injured leg compared to the contralateral leg. Individuals with a previous hamstring strain showed shorter BFlh fascicles, a greater PA, a lower BFlh FL at contraction and ST FL compared to non-injured athletes. No other changes in the muscle size and architecture of the hamstrings in the injured leg compared with the contralateral leg or uninjured athletes were observed.

### 4.1. Injured vs. Uninjured Limb

The results of this metanalysis showed no differences in FL, PA (Figure 2), MT (Figure 3) and volume (Figure 4) in the two limbs in previously injured athletes. Hence, it is highly unlikely that differences in the muscle size or architecture between the two limbs might account for previously reported strength and flexibility differences [8]. There are two alternative explanations for these observations. First, it could be argued that the development of a scar in the injured area [14] as well as the reduced mobility of the athlete following injury do not influence the size and architecture of the injured limb. Sanfilippo et al. [6] reported an immediate reduction in muscle CSA after injury, which was reduced markedly after 6 months. This is most likely due to the healing process of the trauma after injury. An alternative explanation may be that injury influences the muscle–tendon architecture of both limbs (see next section).

The best-evidence synthesis analysis showed that there is moderate evidence of a lower BFlh FL at contraction compared to the contralateral leg (Table 3). Hence, it appears that hamstring injury reduces the amount of fascicle shortening during contraction. This may be related to reduced neural inhibition [17] and a stiffer tendon/aponeurosis of the injured muscle, as a result of injury [18]. Therefore, even though the injured muscle does not display evident changes in FL, PA and muscle size at rest, there is evidence of an altered mechanical function of this muscle during contraction. This might also explain the lower strength deficit observed in the injured limb relative to the contralateral limb injury [18].

Based on the best-evidence analysis, the injured leg showed similar aponeurosis size, but a greater tendon volume compared to the contralateral leg (Table 3). These results are based on limited or moderate evidence (owing to one study per parameter) and therefore, they should be treated with caution. Nevertheless, it appears that the localized responses in muscle–tendon tissue that occur due to injury probably do not influence aponeurosis size. The greater tendon volume may indicate a greater stiffness of the injured muscle–tendon unit [37], which may explain the greater fascicle shortening of the injured muscle upon contraction [24]. However, Silder et al. [37] reported that changes in tendon volume at rest are not related to strength deficiencies that are observed in the injured compared to the contralateral leg. These contradictory observations illustrate that injury has an influence on the mechanical properties of the muscle–tendon unit, but this may not accompany changes in muscle strength.

### 4.2. Injured vs. Uninjured Groups

With respect to the second question of the present study, the metanalysis indicated that injured athletes show a lower BFlh FL than uninjured ones (Figure 5). The difference in the magnitude of the SMD in BFlh FL between injured athletes and controls was characterized as “medium” with very low heterogeneity in the included studies. Given that most studies that examined BFlh FL were ranked as high quality (Table 1), it seems highly probable that athletes with a previous hamstring strain have shorter BFlh fascicles than non-injured ones. 

Owing to the retrospective nature of the examined studies, the mechanism that explains these observations is unclear. One explanation may be that fascicle shortening is an adaptation of muscle to injury that influences both legs. Nevertheless, the mechanisms that lead to this adaptation are unclear. Timmins et al. [18] commented that fascicle shortening is a response to the reduced excursions of the muscle in the early stages of rehabilitation. This has been linked with a reduced participation in sports [6] as well as the presence of neural inhibition of the hamstrings following injury [6,9,10,35,38,39,40]. The reduced neural activation has been noted during slow eccentric contractions in previously injured athletes compared with controls, even after they returned to pre-injury levels of competition [9,10,17,36]. This mechanism, if present, results in a reduction in the FL of the contralateral leg muscle as well as in other muscles, such as the ST [20] (Table 4). Since there are no comparisons of muscle architecture before and after injury, an alternative explanation may be that in the general population, some athletes have shorter fascicles (of both legs) relative to others. This is supported by prospective data that show that players with shorter fascicles are at a greater risk of sustaining an injury [41]. Further research is necessary to verify these observations.

Given the shorter fascicles of their injured BFlh and an unaltered PA (Table 4), one might expect that individuals with a previous hamstring strain would have a lower volume, thickness or CSA of their injured hamstring compared to controls. However, the results of the best synthesis analysis suggested otherwise (Table 4). Several factors should be taken into consideration when interpreting this finding. First, the studies that examined CSA differences between injured and controls did not measure the FL and PA of the injured muscle [24] and vice versa [19]. Second, regional variations in muscle–tendon morphology may influence the measured FL, PA or CSA [42,43], for example, measurement of CSA from the middle muscle belly may mask atrophies that occur in the proximal or distal part of the muscle [44]. Experiments that compare FL, PA and volume or CSA measurements between injured and controls will be able to provide more accurate information regarding the influence of injury on muscle size.

The present review showed moderate evidence for no differences in aponeurosis morphology between athletes with a history of hamstring strain and controls (Table 4). Since evidence was provided by only one study [24] and given the variability of aponeurosis width and size measurements, further research is necessary before conclusions on the relation between BFlh injury and aponeurosis size can be made.

There are several implications of the present findings. From a mechanical point of view, the shorter BFlh FL and greater PA of the injured BFlh indicates that for the same hamstring lengthening movement, the shorter fascicles would be overstretched relative to longer fascicles [23,41,45,46]. On this occasion, the muscles operate mostly on the descending limb of their force–length relationship; consequently, when they actively lengthen, they are weaker and they may experience greater microscopic damage [5,38]. Based on the present study, therefore, hamstring strain rehabilitation should include exercises that promote fascicle lengthening not only for the injured muscle but also for the contralateral one. If an assessment of the architecture is applied, then this should be performed during contraction conditions and not at rest. Exercises that promote hypertrophy of the injured muscle (as reflected in muscle volume, thickness or CSA) may not be particularly useful for restoring muscle function. Finally, this study indicates that regular screening of athletes for shorter BFlh fascicles may assist in injury prevention, especially for those athletes who have a history of a previous injury.

There are several limitations that may influence the conclusions drawn from this metanalysis. First, we examined studies that contain data from participants who had previously sustained a hamstring injury. This experimental (cross-sectional) design does not permit us to decide whether the reported deficits were the cause of injury or the result of injury. Studies that use prospective experimental designs and follow the muscle–tendon architecture prior to and after injury would provide essential information on the relation between injury and muscle architecture. Second, the number of studies for some muscles (SM, BFsh) and variables (PCSA) is small or absent (Table 1). It is also apparent that evaluation of the muscle architecture during contraction may provide more information on the influence of injury on the muscle–tendon unit function. Third, most studies examined BFlh injuries (Table 1), which occur mostly during sprinting [47]. It is not certain that injuries in other hamstrings, such as ST or SM [47] would have similar effects on the muscle–tendon architecture. If the exact muscle that is getting injured and the injury conditions are not identified, then it is difficult to relate the cause (injury) with specific changes in the muscle–tendon architecture. Further, rehabilitation protocols may vary amongst studies and this can also influence architecture measures after injury.

## 5. Conclusions

Τhe present systematic review and metanalysis showed that athletes with a previous BFlh injury show no signs of altered size or architecture in their injured limb compared to their non-injured one when they are at rest. Moderate evidence supports a greater fascicle shortening during contraction in the injured compared to the uninjured leg. By comparison to healthy participants, those who have a history of hamstring injury are likely to have shorter BFlh fascicles while there is no information about differences in the size and architecture of other muscles. Exercises that promote fascicle lengthening of both muscles may be beneficial for preventing hamstring re-injury.

## Figures and Tables

**Table 1:** Risk of bias assessment.

Study	1	2	3	5	6	7	10	11	12	16	18	20	21	25	27	28	29	Total	%	Quality
Avrillon et al. [22]	1	1	1	2	1	1	1	0	0	1	1	1	1	0	0	1	1	14	70	High
Bourne et al. [33]	1	1	1	1	1	1	1	0	0	1	1	1	1	0	1	2	0	14	70	High
de Lima-E-Silva et al. [19]	1	1	1	2	1	1	1	0	0	1	1	1	1	0	0	2	0	14	70	High
Freitas et al. [24]	1	1	1	2	1	1	1	0	0	1	1	1	1	0	1	2	0	14	70	High
Mühlenfeld et al. [34]	1	1	1	2	1	1	1	0	0	1	1	1	1	0	0	2	2	16	80	High
Nagano et al. [35]	1	1	1	1	1	1	0	0	0	1	0	1	1	0	0	0	0	9	45	Low
Nin et al. [20]	1	1	1	1	1	1	0	0	0	1	1	1	1	0	0	0	0	10	50	Low
Sanfilippo et al. [6]	1	1	1	2	1	1	1	0	0	1	1	1	1	1	0	2	2	17	85	High
Silder et al. [23]	1	1	1	0	1	1	1	0	0	1	1	1	0	1	0	2	1	13	65	Low
Timmins et al. [18]	1	1	1	1	1	1	1	0	0	1	1	1	1	1	1	2	1	16	80	High
Timmins et al. [21]	1	1	1	2	1	1	0	0	0	1	1	1	1	0	1	1	0	13	65	Low

**Table 2:** General characteristics of the included studies.

Study	Participants (Age)	Injured Muscle	Time from Injury	Muscle	Architecture	Imaging Technique
Avrillon et al. [22]	17 M elite sprinters and long jumpers (26.3 ± 5.5 yrs)	BFlh	98.2 ± 53.3 days	BFlh, BFsh, ST and SM	PCSA, MV, FL *, PA *	MRI and US
Bourne et al. [33]	10 M recreationally active, (21.6 ± 1.9 yrs)	7 BFlh, 2 ST, 1 SM	within the previous 24 months	Hamstrings	ACSA	MRI
de Lima-E-Silva et al. [19]	80 M football players I: 20 (22.10 ± 3.65 yrs), CG: 60 (19.25 ± 2.40 yrs)	(Not specified)	Prior football season	BFlh	FL	US
Freitas et al. [24]	40 M professional football players I: 9 (28.6 ± 5.2 yrs), CG: 31 (23.3 ± 4.3 yrs)	BFlh	1.41 ± 1.04 years	BFlh	MV, ACSA, aponeurosis	MRI
Mühlenfeld et al. [34]	20 M football players (25 ± 4 yrs)	Whole group	45 ± 15 h	Hamstrings	MV	MRI
Nagano et al. [35]	6 M track and field sprinters (20.3 ± 0.8 yrs)	Not specified	2–8 weeks	BFlh, ST	MT	US
Nin et al. [20]	15 M athletes, IG:5 (22.8 ± 1.9 yrs) CG: 10 (23.2 ± 2.1 yrs)	BFlh	18 months	BFlh, ST	MT, PA, FL	US
Sanfilippo et al. [6]	IG: 22 M and F recreational athletes (24 ± 9 yrs)	16 BFlh, 4 SM, 2 SM	26 (17–49 days)	BFlh, BFsh ST	MV	MRI
Silder et al. [23]	IG: 18 M and F athletes (24 ± 9 yrs)	BFlh	5–13 months	BFlh	Tendon volume	MRI
Timmins et al. [18]	20 M recreationally active and 16 elite athletes IG:16 (23.7 ± 3.3 yrs), CG: 20 (26.1 ± 7.4 yrs)	BFlh	18 months	BFlh	MT, PA, FL	US
Timmins et al. [21]	30 M elite Australian Football IG:12 (22.9 ± 2.6 yrs), CG: 18 (23.5 ± 3.9 yrs)	BFlh	12 months	BFlh	FL, MT, PA	US

M: Male; F: Female; I: injured; CG: Control Group; HSI: Hamstring Strain Injury; BFlh: Biceps Femoris long head; BFsh: Biceps Femoris short head; SM: Semimembranosus; ST: Semitendinosus; PCSA: Physiological Cross-Sectional Area; MV: Muscle Volume; FL: Fascicle Length; PA: Pennation Angle; ACSA: Anatomical Cross-Sectional Area; MT: Muscle Thickness; US: Ultrasound; MRI: Magnetic Resonance Imaging. * Calculated for BFlh, BFsh and SM.

**Table 3:** Results of best evidence synthesis of studies that compared muscle architecture between injured and non-injured limb in participants with previous hamstring injury.

Parameter	Risk Bias	Diff	Evidence Level	Studies
Fascicle Length				
SM, BFsh	Low	↔	Moderate	Avrillon et al. [22]
ST	High	↔	Limited	Nin et al. [20]
BFlh FL/muscle length	Low	↔	Moderate	de Lima-E-Silva et al. [19]
BFlh FL, BFlh FL/muscle thickness at 0, 25, 50 and 75% MVC	Low	↓	Moderate	Timmins et al. [18]
Pennation Angle				
SM	Low	↔	Moderate	Avrillon et al. [22]
BFsh	Low	↔	Moderate	Avrillon et al. [22]
ST	High	↔	Limited	Nin et al. et al. [20]
BFlh at 25, 50 and 75% MVC	Low	↑	Moderate	Timmins et al. [18]
Muscle thickness				
BFlh, ST during contraction	High	↔	Limited	Nagano et al. [35]
BFlh at 25, 50 and 75% MVC	Low	↔	Moderate	Timmins et al. [18]
Physiological Cross-sectional area				
ST, SM, BFlh, BFsh	Low	↔	Moderate	Avrillon et al. [22]
ST/Hamstrings	Low	↔	Moderate	Avrillon et al. [22]
SM/Hamstrings	Low	↔	Moderate	Avrillon et al. [22]
BFlh/Hamstrings	Low	↓	Moderate	Avrillon et al. [22]
Anatomical Cross-sectional area				
BFlh	Low	↔	Moderate	Freitas et al. [24]
Hamstrings	Low	↔	Moderate	Bourne et al. [33]
Muscle Volume				
SM	Low	↔	Moderate	Avrillon et al. [22]
Hamstrings	Low	↔	Moderate	Mühlenfeld et al. [34]
Tendon/aponeurosis				
BFlh aponeurosis Width, Area, Volume	Low	↔	Moderate	Freitas et al. [24]
BFlh tendon Volume	High	↑	Lim	Silder et al. [23]

Diff: difference between injured and contralateral leg muscle; ↓: injured limb lower or ↑ greater or ↔ no different compared to the contralateral leg; SM: Semitendinosus; BFsh: Biceps Femoris short head; BFlh: Biceps Femoris long head; ST: Semitendinosus; FL: Fascicle Length; MVC: Maximum Voluntary Contraction.

**Table 4:** Results of best evidence synthesis of studies that compared muscle architecture between participants with previous hamstring injury and controls.

Parameter	Risk Bias	Diff	Evidence Level	Studies
Fascicle Length				
BFlh FL/muscle length	Low	↓	Moderate	de Lima-E-Silva et al. [19]
BFlh FL/muscle thickness, BFlh FL at 25, 50 and 75% MVC	Low	↔	Moderate	Timmins et al. [18]
ST	High	↓	Limited	Nin et al. [20]
Pennation Angle				
BFlh at 25, 50 and 75% MVC	Low	↔	Moderate	Timmins et al. [18]
ST	High	↔	Limited	Nin et al. [20]
Muscle thickness				
ST	High	↔	Limited	Nin et al. [20]
BFlh at 25, 50 and 75% MVC	Low	↔	Moderate	Timmins et al. [18]
Volume and Cross-sectional area				
BFlh	Low	↔	Moderate	Freitas et al. [24]
Aponeurosis				
BFlh width, area, volume	Low	↔	Moderate	Freitas et al. [24]

Diff = difference between groups; ↓ = injured lower compared to control group; ↑ = injured greater compared to control group; ↔ = injured not different compared to control group; SM: Semitendinosus; BFsh: Biceps Femoris short head; BFlh: Biceps Femoris long head; ST: Semitendinosus; FL: Fascicle Length; MVC: Maximum Voluntary Contraction.

## Data Availability

Data supporting reported results will be available at Mendeley.
